# Sensorimotor maps can be dynamically calibrated using an adaptive-filter model of the cerebellum

**DOI:** 10.1371/journal.pcbi.1007187

**Published:** 2019-07-11

**Authors:** Emma D. Wilson, Sean R. Anderson, Paul Dean, John Porrill

**Affiliations:** 1 School of Computing and Communications, Lancaster University, Lancaster, United Kingdom; 2 Department of Automatic Control and Systems Engineering, University of Sheffield, Sheffield, United Kingdom; 3 Department of Psychology, University of Sheffield, Sheffield, United Kingdom; Western University, CANADA

## Abstract

Substantial experimental evidence suggests the cerebellum is involved in calibrating sensorimotor maps. Consistent with this involvement is the well-known, but little understood, massive cerebellar projection to maps in the superior colliculus. Map calibration would be a significant new role for the cerebellum given the ubiquity of map representations in the brain, but how it could perform such a task is unclear. Here we investigated a dynamic method for map calibration, based on electrophysiological recordings from the superior colliculus, that used a standard adaptive-filter cerebellar model. The method proved effective for complex distortions of both unimodal and bimodal maps, and also for predictive map-based tracking of moving targets. These results provide the first computational evidence for a novel role for the cerebellum in dynamic sensorimotor map calibration, of potential importance for coordinate alignment during ongoing motor control, and for map calibration in future biomimetic systems. This computational evidence also provides testable experimental predictions concerning the role of the connections between cerebellum and superior colliculus in previously observed dynamic coordinate transformations.

## Author summary

The human brain contains a structure known as the cerebellum, which contains a vast number of neurons–around 80% of the total ~90 billion. We believe the cerebellum is involved in learning motor skills, and so is vitally important for accurately controlling the movements of our body, amongst other things. However, like most regions of the brain, we still do not fully understand the role of the cerebellum and evidence for new roles is appearing all the time. One such new role is in the calibration of sensorimotor maps in the brain that link our sensory perception to motor function, such as when a visual stimulus causes a redirect of our gaze. We investigated this problem by connecting a mathematical model of the cerebellar cortical microcircuit to simulated sensory maps in the superior colliculus that are used to control orienting movements. We found the error signal generated by inaccurate orienting movements could be used to accurately calibrate sensorimotor maps, and to allow predictive tracking of moving targets. This finding points to a potentially widespread role for the cerebellum in calibrating the sensorimotor maps that are ubiquitous in the brain and could prove useful in controlling the movements of multi-joint robots.

## Introduction

Evidence for cerebellar involvement in map calibration comes from studies of prism adaption in primates [1, 2] and cerebellar patients [3–6], and from measurements of human brain activity during adaptation [7, 8]. This evidence suggests that "the cerebellum is particularly involved in the realignment process that is necessary to re-establish a correct spatial mapping among visuo-motor and sensorimotor coordinate systems" ([7], p.176). Given the ubiquity of map representations in the brain, such involvement represents a very significant new role for the cerebellum. However, although computational studies have indicated how the cerebellum could form internal models of a wide variety of dynamic processes [9–11], it is unclear how these ideas could be applied to the problem of calibrating maps.

One possible mechanism for map calibration is suggested by electrophysiological studies of collicular maps that are used to guide orienting movements. These maps receive information about target location from multiple modalities [12], and issue motor commands to eyes, head and body depending on the species [13]. In primates and humans the superior colliculus primarily controls saccades that bring the target onto the fovea, and these saccades can be artificially miscalibrated by allowing the target to move during the saccade itself [14]. Accuracy can be relearnt, a process termed saccadic adaptation, provided the relevant region of the cerebellum is intact [15]. Current evidence suggests that the cerebellum can act both downstream of collicular maps, and on the maps themselves [16], consistent with the massive reciprocal connections between the cerebellum and the superior colliculus [17].

In the case of maps combining visual and auditory information, a problem arises when the eyes do not look straight ahead, since the head-based auditory coordinate frame becomes misaligned with the visual coordinate frame. Recordings from primate superior colliculus indicate that auditory receptive fields are appropriately altered by information about the position of the eyes in the orbit [18]. Similar results were obtained for a combined visual and somatosensory map, when the task was to saccade to a tactile signal delivered to the hand [19]. These results suggest that the superior colliculus receives map-calibration signals that can vary dynamically on a trial-by trial basis. We therefore investigated whether such signals could be in principle be generated by current computational models of the cerebellum.

We used as a basic framework the standard ‘chip’ metaphor of cerebellar function, which has been employed to represent the combination of a homogeneous cerebellar cortical microcircuit with individual microzones having unique external connections [11, 20]. In this framework we constrained the model by requiring the cerebellar microcircuit to be represented in a familiar form, so that the novel feature was the architecture connecting cerebellum and superior colliculus. The familiar form we chose was the basic adaptive filter model of the cerebellar microcircuit [21], a development of the original Marr-Albus theoretical framework that uses the covariance rule to implement the least mean square learning rule for time-varying input signals. This model has been used successfully in a wide variety of sensorimotor contexts [22], and here we investigated whether it could be applied without change to the very different computational problem of calibrating a topographic map driving an orienting response.

We determined whether the model was capable of acquiring two competencies, first correcting a unimodal map that has become distorted and secondly resolving mismatches between modalities in a multimodal map. In addition, since the algorithm we chose naturally results in maps which are predictive, we examined how the cerebellum could be used to calibrate prediction of the future position on the map of a moving target. This competence has been demonstrated for the auditory tectal map in the owl [23] and a colliculus-related map in cat [24], and is consistent with the demonstrated role of the superior colliculus or optic tectum in prey catching in a number of species [25–29].

## Results

### Overview of architecture for map calibration

**Fig 1:**
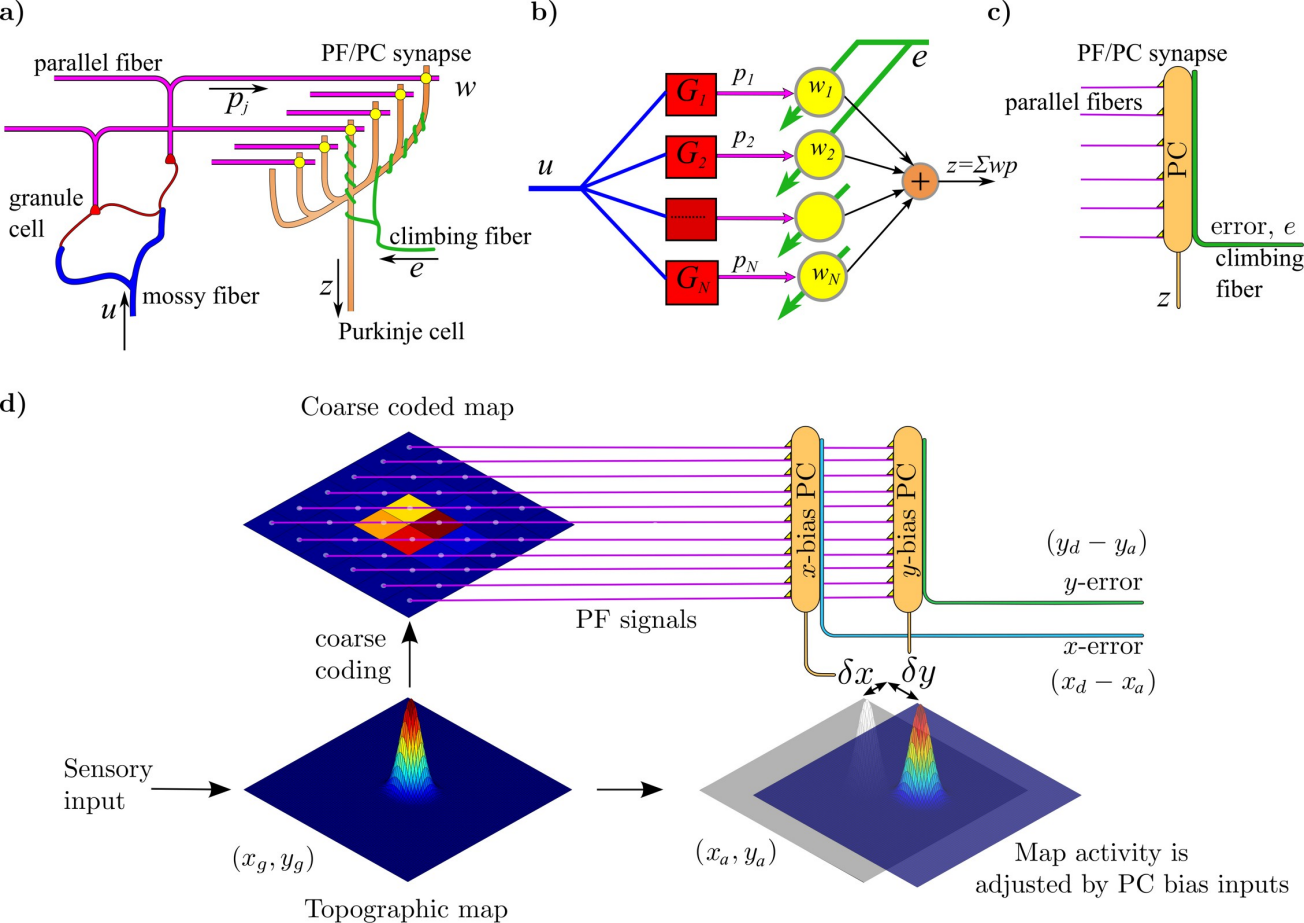
Cerebellar-collicular circuitry for calibration of unimodal sensory maps. a) Simplified diagram of cerebellar cortical microcircuit, in which mossy-fibre inputs *u* are recoded in the granular layer to produce parallel-fibre signals *p_j_*. These signals influence the simple spike firing *z* of Purkinje cells via the synapses *w*. Purkinje cells also a receive a climbing-fibre input *e*. b) Interpretation of the microcircuit as an adaptive filter. In the adaptive filter model, each microzone has two inputs (climbing fibre, mossy fibre) and a single Purkinje cell output. Processing in the granular layer is represented by a set of fixed filters *G_1_ … G_N_* whose outputs *p_1_ … p_N_* are weighted by *w_1_ … w_N_* where the weights correspond to the efficacies of the synapses between parallel fibres and Purkinje cells. Purkinje cells linearly sum the weighted parallel-fibre signals to produce their simple-spike output *z* = *Σw_i_p_i_*. The climbing-fibre input *e* acts as a teaching or error signal that alters the weights *w_1_ … w_N_* using the covariance learning rule *𝛅w_i_* = *-β<ep_i_>*. c) Compact schematic used to illustrate the parallel fibre (PF), Purkinje cell (PC), and climbing fibre part of the circuit in subsequent diagrams. d) Schematic diagram of the proposed recalibration architecture for a distorted unimodal collicular map. Unimodal sensory signals, corresponding to target locations (*x_d_, y_d_*), are written into the map, which because of the distortion provides an inaccurate estimate of target location (*x_g_, y_g_*) that is used to generate a correspondingly inaccurate orienting response. The map output is also sent to the cerebellum, where it is converted into a coarse-coded, normalised set of parallel-fibre (PF) signals. These are sent to two cerebellar microzones, each represented in the diagram by a single Purkinje cell, that receive climbing fibre inputs that initially signal errors (*x_d_-x_g_, y_d_-y_g_*) in the orienting response. These errors are used to alter PF-PC synapses, generating cerebellar output that shifts the map so that the orienting response is now made to the new location (*x_a_, y_a_*). This process is repeated until the error (*x_d_−x_a_, y_d_-y_a_*) becomes zero. Further details in text.

Fig 1 shows in schematic form the architecture for calibration of a unimodal sensory map, in which the adaptive filter learns to produce dynamic modulating inputs to the map that increase its accuracy. The cerebellar cortical microcircuit is modelled as an adaptive filter [21, 22]. This uses a systems level interpretation, in which each cerebellar microzone has two inputs (climbing fibre, mossy fibre) and a single Purkinje cell output. Such a model has previously been applied in a range of sensorimotor contexts [22]. We keep the same model hardwiring (as described below and in Fig 1A and 1B) to determine if it can still be applied in the very different context of map calibration.

A simplified version of the microcircuit is shown in Fig 1A, in which the mossy-fibre inputs *u* are recoded in the granular layer to produce parallel-fibre signals *p*_*j*_. These signals influence the simple spike firing *z* of Purkinje cells via the synapses *w*. Purkinje cells also a receive a climbing-fibre input *e*. In the adaptive-filter interpretation of this circuit (Fig 1B) processing in the granular layer is represented by a set of fixed filters *G*_*1*_ … *G*_*N*_ whose outputs *p*_*1*_ … *p*_*N*_ are weighted by *w*_*1*_ … *w*_*N*_ where the weights correspond to the efficacies of the synapses between parallel fibres and Purkinje cells. Purkinje cells linearly sum the weighted parallel-fibre signals to produce their simple-spike output *z = Σw*_*i*_*p*_*i*_. The climbing-fibre input *e* acts as a teaching or error signal that alters the weights *w*_*1*_ … *w*_*N*_ using the covariance learning rule *𝛿w*_*i*_ *= -β<ep*_*i*_*>*, which corresponds to the Least Mean Square learning rule [30]. In this form of supervised learning the weights are altered until correlations between presynaptic inputs and output error are removed [30, 31], hence the term decorrelation learning [32]. A compact schematic of the adaptive filter (Fig 1C) is used in subsequent diagrams.

In the simplest version of the architecture the superior colliculus was represented by a single topographic map (Fig 1D). Target locations **x**_**d**_ = (*x*_*d*_,*y*_*d*_) are selected from within a two dimensional grid, then transformed into sensor data which is written into the collicular map, modelled as a square grid with each grid point corresponding to a collicular neuron. The sensor data are generated by a linear sensor model **s**_**d**_ = **Kx**_**d**_, where **K** is a 2 x 2 matrix that defines the sensor model and is determined from the sensor scaling, noise level and rotation of target (e.g. [33]). The sensor data are then written into the topographic collicular map to provide a distributed representation of the target location (Fig 1D). Neurons in the map had receptive field centres (*x*_*i*_,*y*_*j*_), so that if only an individual neuron fired, it would produce an orienting response to the real-world location (*x*_*i*_,*y*_*j*_). It assumed here that the map’s connections to the motor system are fixed, and that neuron centres are assumed to be dense enough to code the target location accurately. A 2D elliptical Gaussian function was used to provide the distributed target position which when sent to the motor system generates an orienting response to the estimated position of the target. For an accurate map this corresponds to the actual position of the target **x**_**d**_, thus bringing the target onto the fovea (in primates) or the area of the mouth (in rodents). The two components are calculated from the distributed firing rates of the collicular neurons (details in Materials and Methods).

The distributed collicular response is also sent to the cerebellum as mossy-fibre input, where it is processed in the granular layer to produce a coarse coded map carried by the parallel fibres (Fig 1D). Coarse coding was used to provide a sparser representation to ensure both an acceptable speed of learning and an acceptable degree of precision. An evenly spaced *k* by *k* grid of Gaussian receptive fields, $G_{P}^{n}$ (where *n* denotes the *n*^*th*^ Gaussian in the *k* by *k* grid) was used to coarse code the topographic map. The activity of each grid point was found by multiplying each Gaussian receptive field by the topographic map activity and summing and normalising.

When the collicular map is correctly calibrated, the target positions estimated by the map are accurate, and so are the orienting movements it generates. In the absence of orienting errors the climbing fibres to the cerebellum will not carry any error signals, and the weights between parallel fibres and Purkinje cells will stay fixed. When the collicular map is inaccurate it generates an erroneous estimate **x**_**g**_ = (*x*_*g*_, *y*_*g*_) of the actual target location **x**_**d**_ = (*x*_*d*_, *y*_*d*_) so that the resulting orienting movement will be in error (**e** = **x**_**d**_−**x**_**g**_). This would be foveation error in the case of saccade generation, or a tactile signal provided by micro-vibrissae in the case of rodent prey acquisition. The cerebellum receives a corresponding error signal via climbing fibres, a signal assumed here to be signed and two dimensional, with axes approximately aligned with horizontal and vertical (x and y directions). This error signal is used to adjust the weights of the synapses between parallel fibres and Purkinje cells so that the output to the superior colliculus sent from the cerebellar cortex via the deep cerebellar nuclei biases the collicular map in order to shift the position of peak map activity (Fig 1D). The simplest way for the cerebellum to act on a topographic map is to assume a 2D output **z** which is fed to all neurons in the map and biases their centre position. That is, for a given sensory map, a cerebellar bias input **z** to a target neuron with centre **x** will make it act as though it has centre **x**+**z**. In effect cerebellar input ‘slides’ map activity across the map by an amount **z** = (*δx*,*δy*). We therefore assume there are 2 biasing microzones for each sensory map, so that map activity can be shifted independently in 2 dimensions. Using a global map shift is a simplification that can be applied when considering single targets. For multiple targets, different regions of the map are likely to require shifting by different amounts. To achieve this, the map could be split into different regions, calibrated by a separate cerebellar zones. We consider single targets to avoid overcomplicating the problem.

We use the notation **x**_**a**_ = (*x*_*a*_,*y*_*a*_) to denote the adjusted target location **x**_**a**_ = **x**_**g**_+**z**. Subsequent orienting errors are calculated from the shifted estimated location **e** = **x**_**d**_−**x**_**a**_ = (*e*_*x*_,*e*_*y*_) (further details in Fig 1).

The bias signal is generated as follows. A weight is associated with each parallel fibre signal. The cerebellar weights to bias the map in the *x*—and *y*–directions are learnt from initial values of zero. As indicated above, the learning rule is given by Δ*w*_*x*_ = −*β e*_*x*_ *P*, Δ*w*_*y*_ = −*β e*_*y*_ *P*, where *e*_*x*_ and *e*_*y*_ are the errors, P the coarse coded parallel fibre signals, and *β* is a learning rate.

### Calibration of single map

**Fig 2:**
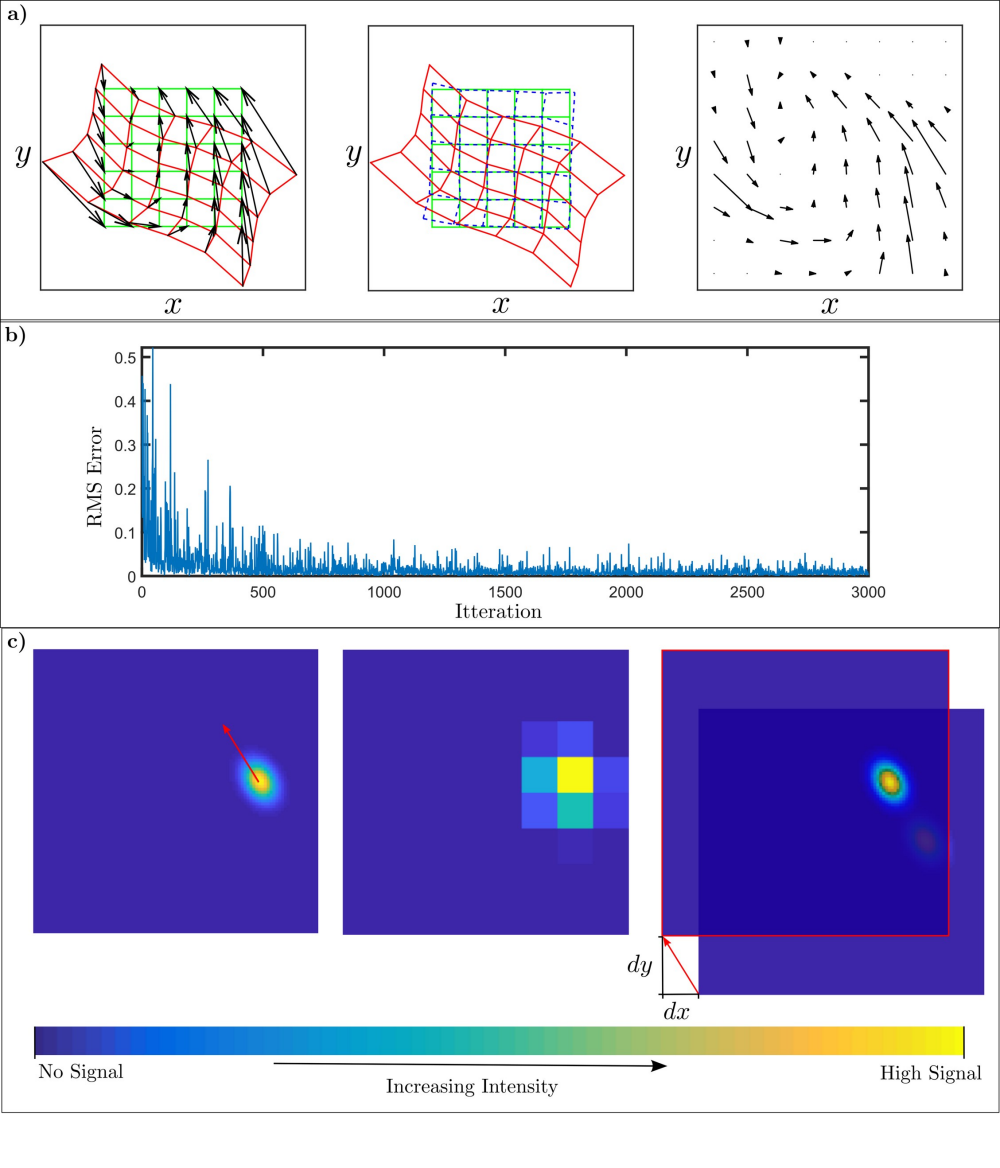
Recalibration of a single target map with curvilinear distortion. **a)** The left hand panel shows an initially accurate map (green line) in the superior colliculus (SC), with artificially induced curvilinear distortion (red line) (details in Methods). The shifts in the map to correct for the distortion are dependent on the location in the map, and are indicated by black arrows. The learnt cerebellar recalibration of the distorted grid (teal line) is shown in the middle panel. The right hand panel shows the combined learnt weights in the x- and y-directions corresponding to each coarse coded parallel fibre signal (weights initially zero). **b)** Time course of recalibration, showing how RMS errors in orienting responses change with number of target presentations. c) Example of learnt dynamic cerebellar recalibration. The left-hand panel shows the shift in the map (red arrow) required to produce an accurate orienting response to the inaccurate target location provided by the distorted map. The centre panel shows the coarse-coded, normalised parallel fibre signals produced by the inaccurate target location. The right hand panel shows that after learning the parallel fibre signals now shift the map by just the required amount to produce an accurate response.

In the first problem we asked the cerebellar-collicular architecture described above to calibrate a unimodal map (green grid in Fig 2A, left-hand panel) that had been distorted as a result of sensor changes (red grid). The nature of the distortion varied with stimulus location, as indicated by the arrows which show the changes to the map that are needed to restore its accuracy. The sensory map after 3000 trials of cerebellar recalibration (blue dashed grid) is shown in the centre panel of Fig 2A, and is very substantially restored to its undistorted from. The right hand panel shows the combined learnt weights in the x- and y-directions corresponding to each coarse coded set of parallel fibre signal (weights initially zero). The time course of the recalibration is shown in Fig 2B, which plots the RMS error of the orienting response against number of stimulus presentations.

The impact of learning maps with a low quality error signal was also investigated by testing a version of the learning rule that simply used the sign of the error signal. Learning with the full signal (Fig 2B) gave RMS errors with mean 0.008 over the last 2500–3000 iterations. When the sign of the error was used this was increased to 0.015. However, both signals substantially restored the map to the undistorted form. The model is robust to reductions in the quality of the error signal, even if it is sign only, learning is little affected.

The details of dynamic recalibration for a particular target location are illustrated in Fig 2C. The shift needed to restore response accuracy to this location is shown as a red arrow on the collicular map image in the left panel. The coarse-coded, normalised parallel fibre signals generated by the inaccurate target location are shown in the centre panel (cf. Fig 1D). At the start of recalibration, each of the weights of these signals (corresponding to the efficacy of the corresponding synapses on Purkinje cells) were zero. After learning the weights had changed to produce a cerebellar output that shifted the map appropriately (Fig 2C, right-hand panel). It is important to emphasise that recalibration by the architecture described above is a dynamic process since the cerebellar bias signal depends on the current target position. This means that, although the whole map receives the same bias signal, the bias signal changes according to the position of the target.

The parallel-fibre representation used here contains enough terms to allow affine recalibrations. In general the complexity of possible re-calibrations depends only on the completeness of the parallel-fibre representation, e.g. radial basis function inputs could generate very general calibrations.

### Calibration of multiple maps

**Fig 3:**
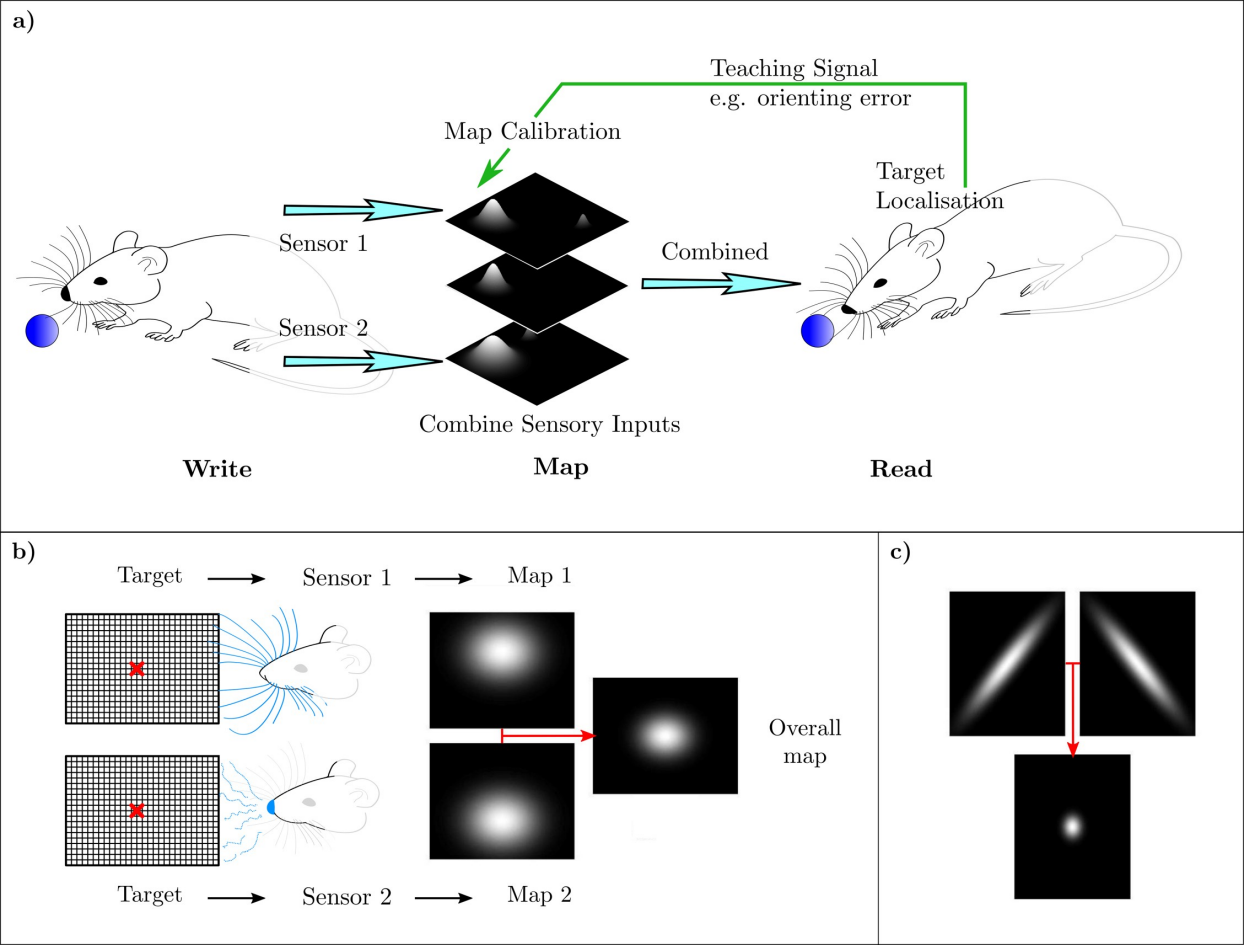
Calibration of combined unimodal maps. a) Individual sensors are assumed to write into 2D unimodal topographic maps, each of which provides a probabilistic representation of target position as shown in Fig 1. The outputs of these unimodal maps are then combined to produce an overall multimodal map, and the position of peak activity on this map drives the orienting response. The problem is how errors in the orienting response can be used to calibrate both unimodal and multimodal maps. b) Combining information from multiple sensors using probabilistic maps can produce a more accurate estimate of location. c) Information between two sensors combines to give a more focused estimate of location. The top plots show individual sensor maps and the bottom the combined map.

The superior colliculus has both unimodal and multimodal maps (e.g. [34]). In the example illustrated in Fig 3, information from a visual and a somatosensory map are combined into a multimodal map that drives the orienting response. If one or both unimodal maps are distorted, the output of the multimodal map produces an inaccurate orienting response. The problem is to use this error information to calibrate all three maps.

**Fig 4:**
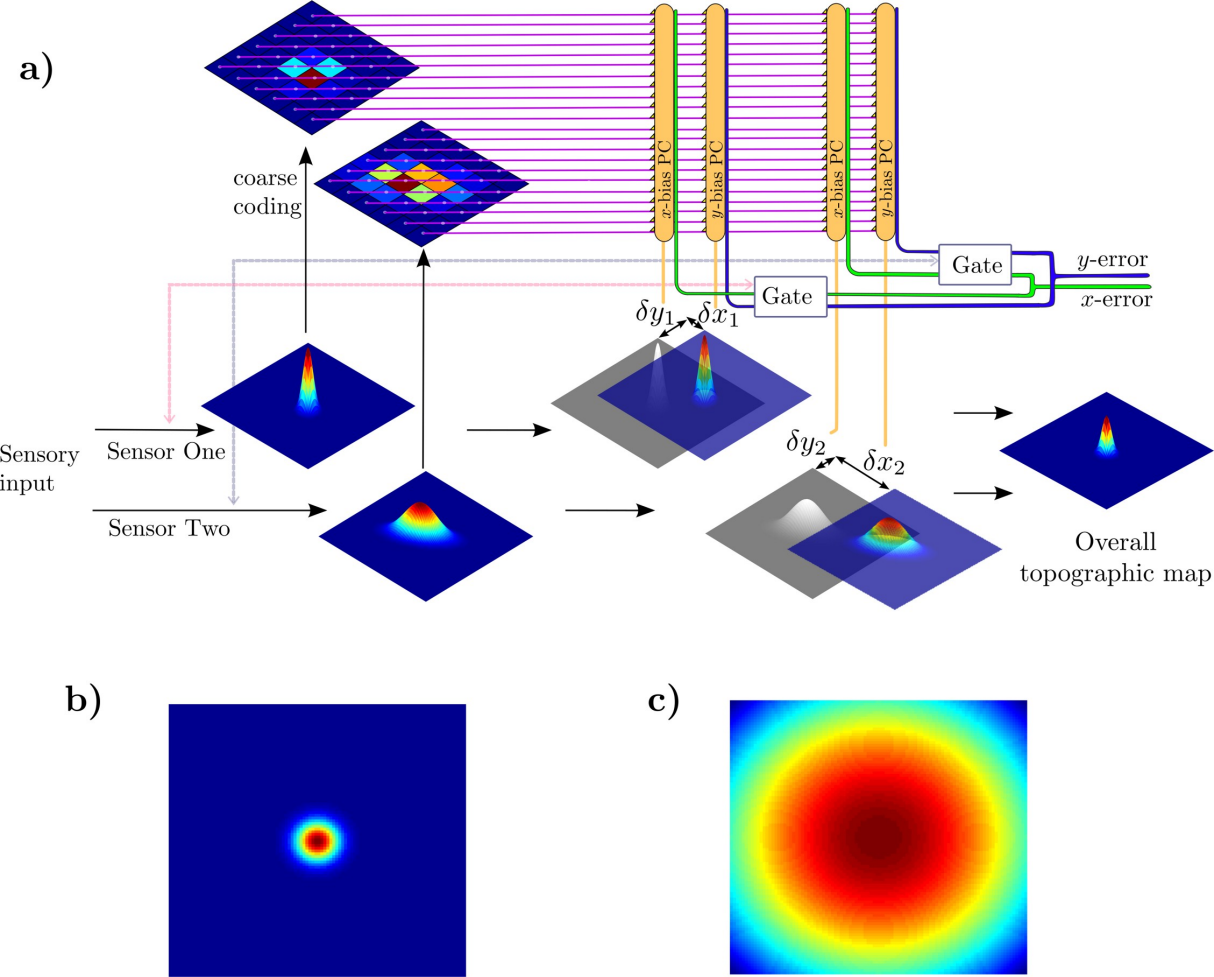
Schematic of architecture for calibrating multiple topographic maps. **a)** The overall topographic map combines information from unimodal maps that are each obtained from a unimodal sensory input. The combined map is used to drive the orienting response, which if incorrect generates an error signal. Parallel fibre signals are a combination of all coarse coded individual sensory maps. Separate Purkinje cells are used to calibrate each unimodal map individually. Gating is introduced to solve credit assignment issues that arise due to the same error signal training all individual sensors. **b)** Map response to target at centre when sensor is not gated (∑ = [0.0225 0; 0 0.0225]). **c)** Map response to target at centre when sensor is gated (∑ = [4.5 0; 0 4.5]). The spread of possible target locations is increased when gating is included.

The architecture used to address this problem (Fig 4A) was an extension of that used for calibrating a single map (Fig 1). For the case of two sensors we assume two sets of PCs, where each set consists of an *x*- bias and *y*-bias PC. Writing undistorted sensory data into each map used linear sensor models as before, where the sensory signals were generated from target locations **x**_d_ by **s1**_**d**_ = **K_1x**_**d**_, **s2**_**d**_ = **K_2x**_**d**_. Both **K_1** and **K_2** were set to the same value to simplify the simulation. The sensor data were then written into the topographic collicular map to provide a distributed representation of the target location as previously, using identical 2D elliptical Gaussian functions. The outputs of the unimodal maps were combined to generate the multimodal map using element by element multiplication of the individual multimodal maps, a method that implements Bayes’ rule (Materials and Methods). Copies of the distributed neuronal responses in the unimodal maps were also sent to the cerebellum as parallel-fibre inputs (Fig 4A). Coarse-coded parallel-fibre signals for each map were generated as before, with the same values for the parameters for each set. The total parallel fibre signal *P* is thus a vector consisting of the values of *P*_*1*_ at each grid point and *P*_*2*_ at each grid point.

When one or both maps were distorted, the output of the multimodal map produced an inaccurate orienting response. In the first method tried for calibrating the unimodal maps, this erroneous response was used to bias the unimodal maps, just as in Task 1 where there was only a single map.

**Fig 5:**
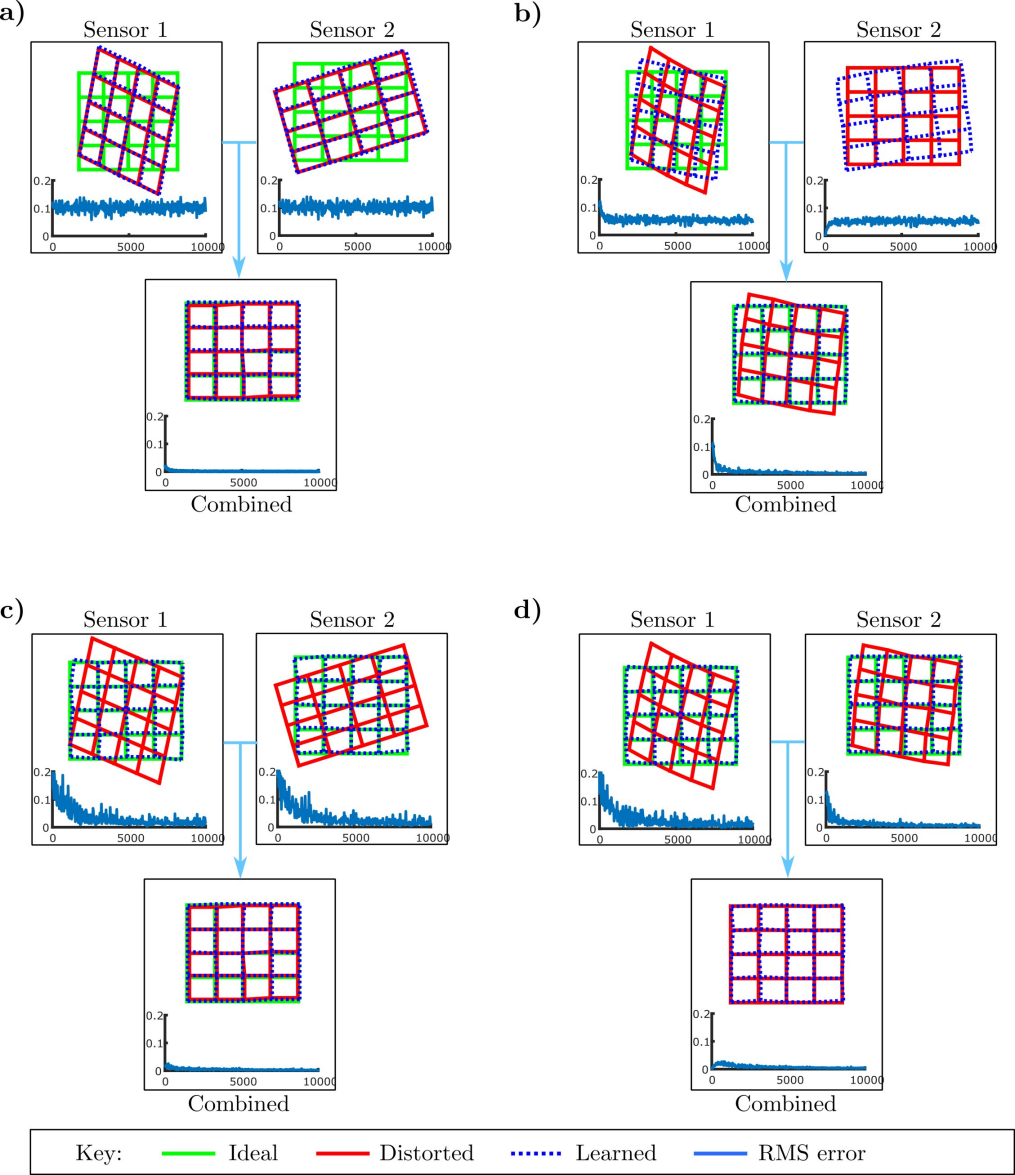
Calibrating multiple unimodal maps: Role of gating. **a)** Demonstration of the credit attribution problem for multiple sensors. Sensors are miscalibrated in such a way that their errors cancel. If all sensor modules are trained by the same overall error signal the individual sensors will not learn even though they are inaccurate. **b)** One sensor has zero error, but the overall error is non-zero. When all sensor modules are trained by the same error signal any behavioural error is necessarily attributed to all sensors and so the individual sensors are forced to learn even if they are accurate. **c)** When errors are gated (details in text) both maps are calibrated even though their errors originally cancelled (panel a). **d)** Gating also prevents an accurate map from being altered (panel b). In both cases the credit attribution problem is solved.

Application of this simple method revealed a fundamental calibration ambiguity. Since estimated target position is a weighted combination of individual map estimates, multiple sensors can be miscalibrated in such a way that their combined errors cancel on average (Fig 5A).

In principle this ambiguity can be resolved if the sensors have varying accuracies, because the relative weightings of different sensors will vary so that cancellation cannot be exact. However, the learning architecture above cannot utilise this information about sensor accuracy, because all sensor calibration modules are trained by the same error signal (from the combined, single map) and so any behavioural error is necessarily attributed to all sensors. This generates a credit attribution problem: since any error is attributed to all sensors, a sensor is forced to learn even when it is accurate (Fig 5B).

The required teaching signal, calculated theoretically by the method of gradient descent, is target error inversely weighted by sensor accuracy. But even in simple cases this requires detailed information about sensor accuracy to modulate the target error signal, and is therefore biologically implausible. A more plausible solution would use available sensory signals as teaching signals.

#### Sensory gating of CF teaching signal

We propose the following adjustment to the basic architecture to implement a simplified credit attribution algorithm, which is in keeping with our principle that there are to be no *ad-hoc* changes to basic microcircuit connectivity or learning rule: When a sensor fails to detect the target the teaching signal is ‘gated’ (Fig 4A) so that no climbing-fibre teaching signal is passed to the relevant bias module. This simplified mechanism is plausible in a biological implementation, and can be shown to converge if sensors have independent non-zero drop-out rates. Here detection failure on a proportion of trials was ensured by altering the sensor property **Σ** (Methods) so that the sensory map gives a very large spread of possible target locations during gating (simulating failure to detect a target, Fig 4B and 4C). This method enables accurate calibration of individual sensors in the case when the combined map errors cancel (Fig 5C), and means that accurate individual sensors are not miscalibrated (Fig 5D). It should be noted that olivary error gating has been observed in motor systems [35].

#### Sensor specific noise

The cerebellar chip philosophy requires that the PF input should be an undifferentiated ‘bus’ of sensorimotor context information and the learning rule should guarantee that only relevant information is used at the synthesis stage. When two sensory maps are being calibrated, this means that the Purkinje cells that bias sensory map 1 receive parallel-fibre signals from map 2, and vice versa (Fig 4A). The cross-talk synapses from these parallel fibres potentially allow sensors to calibrate each other, which can result in miscalibration where two miscalibrated maps cancel their effects on estimated target position.

This potentially serious problem is actually an artefact of the simulation details. In the simulations described above, sensors produce an activity map which encodes sensor accuracy, but in fact no sensor noise was included in these simulations. We therefore introduce independent Gaussian sensory noise e.g. **s1**_**d**_ = **K**_**1 x**_**d**_+*N(0,σ)* alongside gating to remove parallel fibre cross talk synapses (Methods). This sensor noise produces target errors which are only correlated with one sensory input, so that covariance learning guarantees that synapses carrying cross-talk are driven to silence (provided there is gating to break the symmetry between sensors–sensory noise alone without gating did not lead to accurate calibration of individual sensors).

**Fig 6:**
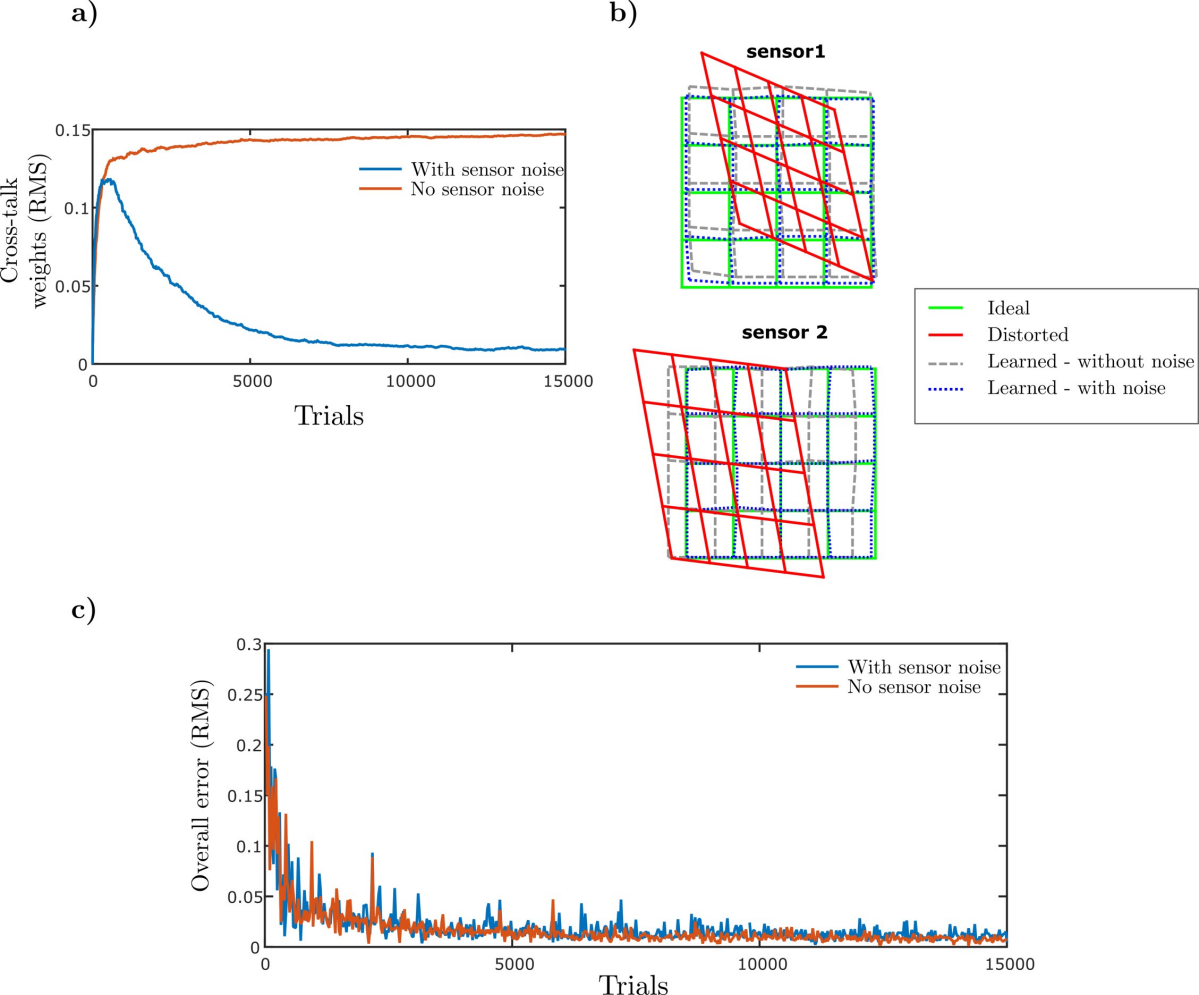
Sensor cross-talk is eliminated when independent sensor noise is present. a) Cross talk weights (given as RMS values) over iterations. When independent sensor noise is present, the cross-talk weights are driven toward zero. b) Individual sensor calibration with and without independent sensor noise. Independent sensor noise eliminates errors in individual sensor calibrations that arise due to cross-talk. The calibrated results are plotted for the case when the input from the other sensor is set to zero. c) Overall RMS errors when both sensors are on and stable for the case when independent sensory noise is included.

The effects of adding noise are shown in Fig 6. The weights of cross-talk synapses are markedly reduced (Fig 6A), with little effect on orienting accuracy (Fig 6B and 6C). It can be seen that there are now two time scales for cerebellar learning (Fig 6A and 6C): a fast time scale in which behavioural errors become small but not optimal and a slower time scale in which synaptic weights are driven to their optimal values.

### Predictive recalibration

Map calibration is often regarded as a static, target independent process. The architecture used here, however, implements a dynamic process since the cerebellar bias signal depends on the current target position. This means that, although the whole map receives the same bias signal, the bias signal changes according to the position of the target. This allows position dependent curvilinear recalibration using a single biasing output as illustrated in Figs 1 and 2. The dynamic formulation turns also leads to a natural implementation of predictive calibration. This is because in the adaptive filter the granular layer is assumed to act as an information processing reservoir, so that the parallel fibres carry information not only about current mossy fibre inputs, but also about the history of those inputs [22]. If we idealise this process by adding further parallel fibre inputs to the biasing microzones which contain the coarse coded map information filtered by leaky integrators at a range of time scales, then, in the presence of delay in either the sensory or motor systems, the adaptive filter learns to predict target position so as to acquire the target accurately.

**Fig 7:**
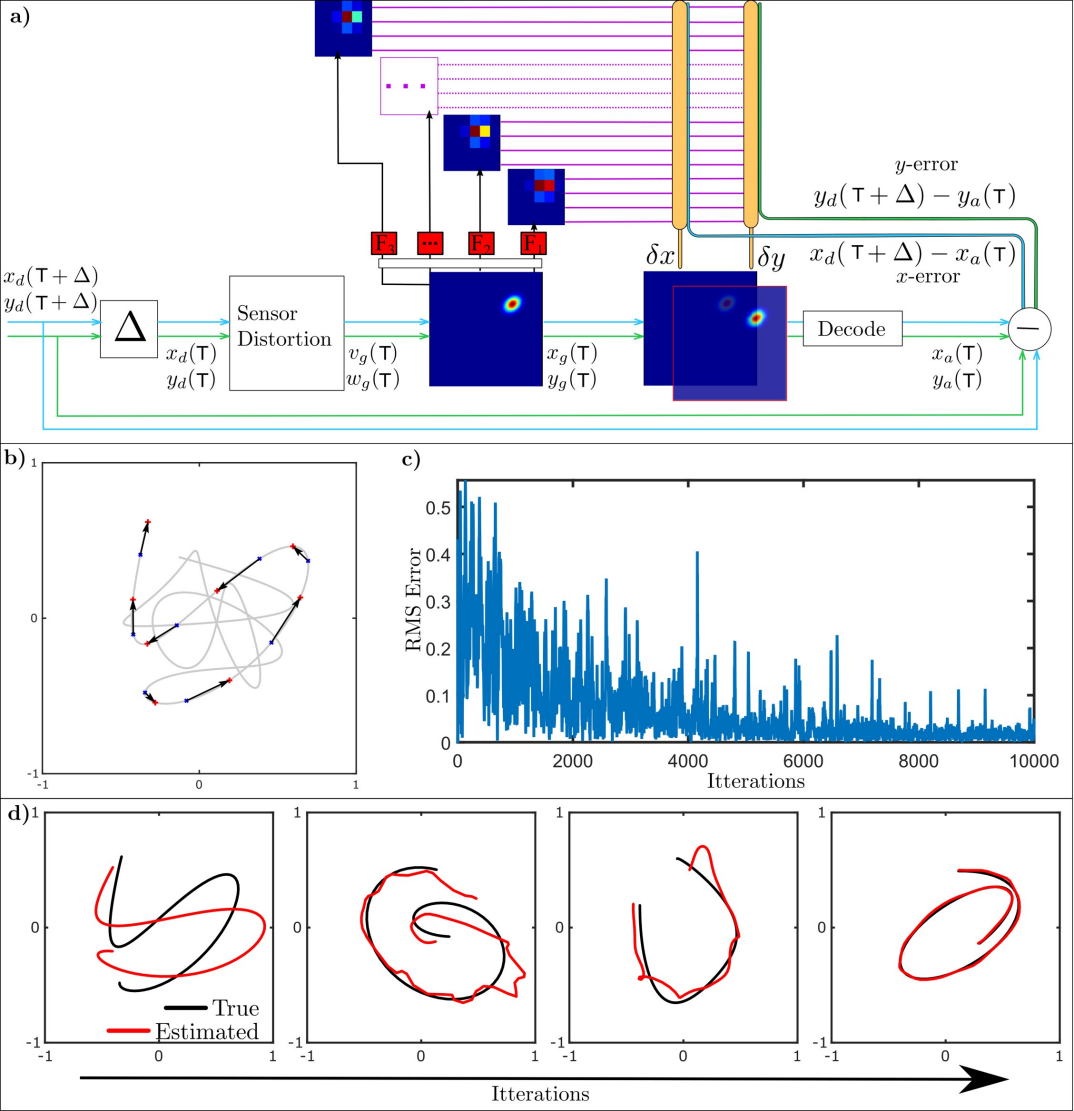
Predictive calibration of a single target map with curvilinear distortion. **a)** Schematic diagram of the proposed recalibration architecture for predictive recalibration. The desired target position is delayed and distorted before writing into a topographic map. Parallel fibre (PF) signals are the filtered outputs (here a bank of 3 leaky integrator filters are used) of a coarse coded, normalised topographic map. **b)** Representation of target trajectory before distortion with 5 sample delay. Examples of the differences between the desired (represented by a red +) and delayed (represented by blue x) targets are indicated by an arrow. The velocities of the example target trajectory ranged from (-1.61,-1.82) units/sec to (1.74, 1.72) units/sec. **c)** RMS errors over iterations when learning to track a target using delayed and distorted sensory information. **d)** Cerebellar learning to predictively recalibrate delayed, distorted signals and estimate the target location. Over iterations, the estimated target trajectory learns to track the desired target trajectory.

Fig 7A illustrates this predictive architecture and Fig 7B, 7C and 7D show the results when applied to a target which moves along a smooth curve (Methods) whose position is both distorted by miscalibration and delayed by sensory processing with respect to the raw sensory input. The algorithm can be seen to successfully reduce mean square acquisition error (Fig 7C), and both remove the distortion and shift the target peak at its predicted position (Fig 7D). There are two time scales involved in the calibration process. Learning the weights (or corrections) is relatively slow and takes place over many iterations. Once the weights are learnt then the application of the corrective signals during dynamic behaviours is fast.

This is only possible because the target motion is predictable; in effect the cerebellum learns an internal dynamic model of target behaviour and uses it to predict future positions. Fig 7 shows that this internal model is optimally adapted to the statistics of the target behaviour, which in this case were bandpassed white noise trajectories chosen as an example of a stochastic motion with an adjustable level of predictability. If the target trajectory only contains low frequencies then prediction is more accurate and uses a simpler internal model based on fewer filter inputs. When higher frequency components are present the trajectory is less accurately predictable and requires a more complex internal model utilising a larger range of filter time-scales.

**Fig 8:**
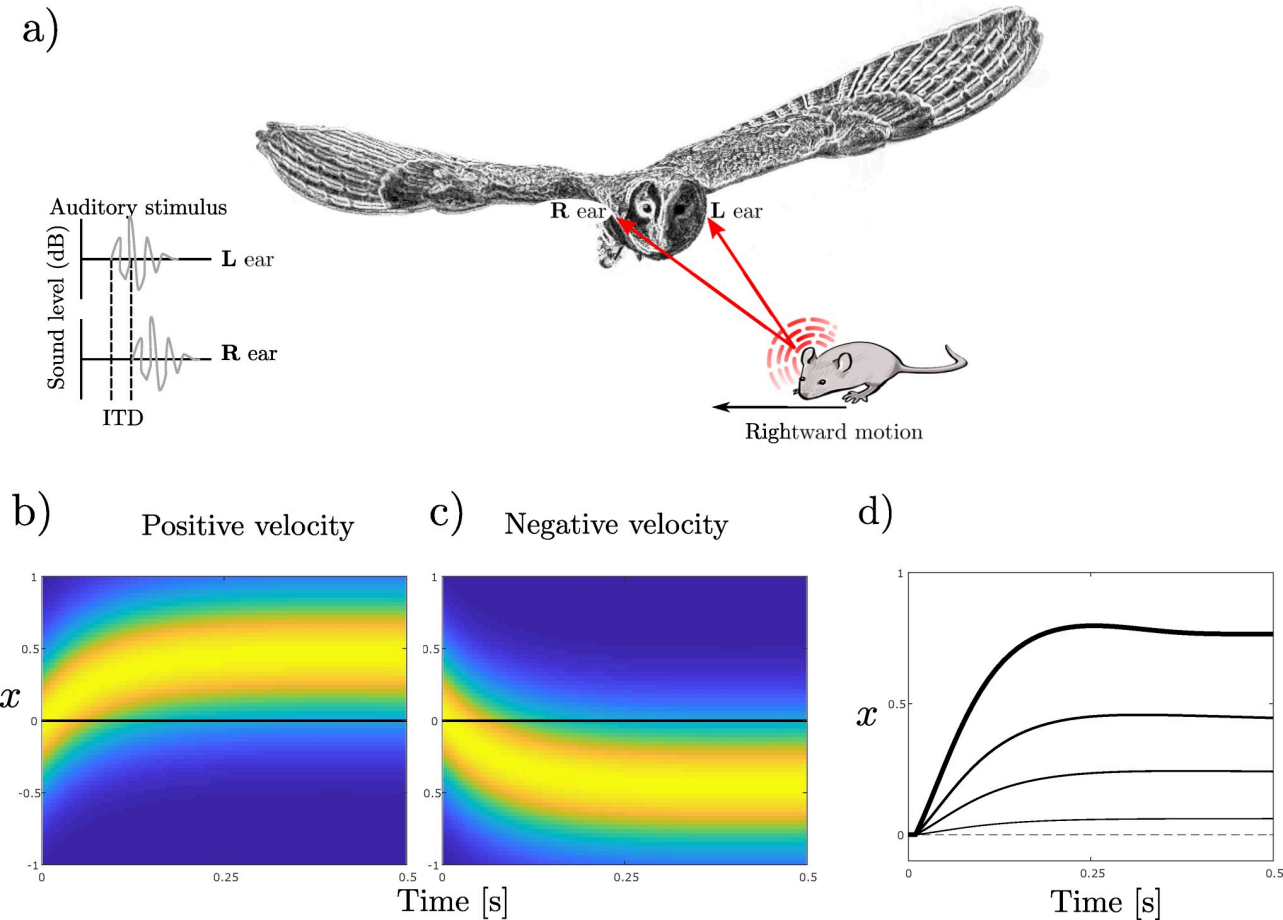
Receptive fields shift in response to a moving target. **a)** Sound waves generated by movements of a mouse are received by the owl’s left and right ears (adapted from [36]). For horizontal positions stimulus location is indicated by interaural time difference (ITD), and stimulus movement by changes in ITD. **b, c, d)** Learnt shifts when tracking a moving target with different velocities using the adaptive filter model. Weights were learnt by tracking a moving target over a single sweep when there was a delay of 100 samples (dt = 5ms) between the estimated and ideal target location (but no sensor distortion). b) Shift of receptive field of target when target is moving with a velocity of 1ms^-1^. c) Shift of receptive filed when target is moving with a velocity of -1ms^-1^. d) Shift for different positive velocities of 0.125, 0.5, 1, 2 ms^-1^(thicker lines correspond to faster speeds). The results are comparable to predictive shifts presented in [23].

Similar predictive shifts in target position have been observed experimentally, for example in the map of auditory space found in the optic tectum of the barn owl [23]. The optic tectum is homologous to the mammalian superior colliculus, and is used by the barn owl to generate orienting movements required for prey capture (Fig 8A). If the prey is moving, then the orienting response must be directed to its predicted not current location, requiring a shift in tectal receptive fields. The nature of such shifts in response to horizontal stimulus movement was examined by manipulating the cue used for localising horizontal position, namely interaural time difference (ITD) using dichotic presentation of sounds through earphones. Sound presentation corresponding to a stimulus location moving at constant velocity elicited receptive field changes corresponding to predicted location (Fig 8B, 8C and 8D).

Consistent with this interpretation, the size of the receptive-field change increases with (virtual) stimulus velocity (Fig 8D). The changes are consistent with a predictive time-lead of ~100 msec, which corresponds to the time taken to complete saccadic gaze shifts produced by electrical stimulation of the tectum [37]. The predictive recalibration architecture (Fig 7A, with simulation parameters provided in the Methods) was able to reproduce this pattern of changes (See Fig 1 in [23] for experimental results). Here no sensor distortion was applied, so the algorithm just learns to account for the delay between the estimated and actual target location. The time scales of the experimental and simulated results differ, however the simulation is not intended to replicate the experiment, but to demonstrate that the adaptive cerebellar filter is able to explain the behaviour seen. Note that an even better correspondence could be obtained if evidence accumulation was added to the salience map write-mechanism, so that sensor inputs were optimally combined over time in the map. This would result in a tighter bound on target location over time, mimicking the behaviour seen in the experimental data.

## Discussion

The form of dynamic remapping investigated here proved effective for calibrating both unimodal and multimodal topographic maps even when these had been distorted in complex ways, and also for using the maps to track predictable stimulus trajectories. These results indicate that the adaptive filter model of the cerebellar microcircuit, which has been widely used in conventional sensorimotor contexts either explicitly or implicitly (e.g. [22]), can in principle be applied to the very different computational problem of calibrating a topographic map driving a motor response.

An important feature of these results is the extent to which they were achieved with no *ad-hoc* changes to basic microcircuit connectivity (Fig 1) and no changes to the covariance learning rule. The climbing-fibre teaching signal was simply related to an available sensory signal, and as far as possible detailed hardwiring of particular parallel-fibre inputs to particular microzones was avoided. All sensors were trained using the same error signal and parallel fibre inputs were an undifferentiated bus of information, the only change was to introduce gating. In principle all potentially relevant input (e.g. raw sensor input) and even irrelevant input could be made available, since a useful characteristic of the adaptive-filter learning rule is that parallel-fibre synapses conveying irrelevant information are driven to silence [38]. This includes Purkinje cell synapses carrying cross-talk between sensors, greatly reducing the need to hard-wire the connectivity of parallel fibre inputs to the cerebellar microzones.

These results provide further evidence of the computational adequacy of the model, and also of the extent to which the cerebellum can be considered to be computationally homogeneous, consisting of a repeating cerebellar microcircuit implementing a single algorithm which is useful in wide range of behaviours (e.g. [11, 20]), justifying its description as a ‘plug-and-play’ cerebellar ‘chip’.

### Biological evidence

Both the cerebellum and superior colliculus have been implicated in saccadic adaptation [14]. The precise nature of that involvement has proved difficult to identify, because saccadic adaptation has turned out to be more complex than it originally appeared, with evidence for different mechanisms being involved depending on whether the adaptation is gain-up or gain-down, short term or long term, or of reactive or voluntary saccades (e.g. [39, 40]). It appears that sensory remapping is likely to be involved in the gain-up adaptation of reactive saccades, and more generally in the adaptation of voluntary saccades (e.g. [41]). In the former case it seems likely that the altered map is within the superior colliculus [16, 33], whereas for the latter spatiotopic cortical maps appear to be implicated [42, 43].

A possible anatomical basis for dynamical cerebellar remapping of maps in the superior colliculus is the extensive projection from the deep cerebellar nuclei to the superior colliculus [17]. However, little is known about the signals sent by these projections, though it has been suggested they may be “involved in correlating the modality maps within the SC” ([17], p.352). There is evidence for tonic excitatory inputs in anaesthetised rats [44–46] that directly influence collicular sensory cells, and affect movements resembling pursuit, but how that influence works during normal behaviour is not understood.

There is good evidence that the cerebellum is involved in the sensory remapping that occurs in prism adaptation [1–5]. The location of the recalibrated maps is however unclear, though event-related FMRI implicates the superior temporal cortex [7]. Adaptation of voluntary saccades has been argued to be similar to prism adaptation [41, 43] and also appears to involve alterations of maps in higher level frameworks than the retinotopic maps in the superior colliculus. The basic framework for map recalibration proposed here should in principle work for such higher-level maps. A necessary requirement for this is the existence of a recurrent architecture involving cerebral cortex rather than the superior colliculus. Evidence for such an architecture connecting multiple cerebellar and cortical areas has been summarised by Ramnani [47].

Overall, the biological evidence appears to be consistent in broad terms with the map calibration scheme proposed here. The next step is to consider more detailed evidence, that could be provided by testing specific predictions generated from the present results.

### Biological predictions

As mentioned in the Introduction, accurate saccades to auditory targets can be made when the eyes are in an eccentric starting position, causing auditory and visual maps to become misaligned [18]. The scheme investigated here predicts that saccadic accuracy to auditory stimuli in this situation will be severely impaired after selective inactivation of cerebellar inputs to the superior colliculus, or of collicular outputs to the cerebellum. It also predicts that this impairment will be accompanied by a loss of the shift in auditory receptive fields that normally results from change in eye position, again as demonstrated by Jay and Sparks [18]

Accurate saccades can also be made to somatosensory targets (stimulation delivered to the hands which are not visible) from different starting positions of the eye [19]. We again predict that saccadic accuracy to these somatosensory stimuli under these conditions will be severely impaired after selective inactivation of connections between cerebellum and superior colliculus, and that this impairment will be accompanied by a loss of the shift in somatosensory receptive fields that normally results from change in eye position [19].

Finally, owls are able to capture moving prey, an ability connected with predictive shifts in the receptive field of auditory neurons in the optic tectum [23]. We predict that selective inactivation of connections between the cerebellum and optic tectum will seriously affect the ability to capture moving prey, and abolish the predictive shifts in auditory receptive fields.

### Implications for cerebellar function

The recalibration mechanisms investigated here may have application to the generic problems of realigning collicular maps when the body moves that were outlined in the Introduction. In the absence of a recalibrating input auditory and visual maps would become misaligned when the head moves (e.g. [18]), as would tactile and visual maps when the hands move (e.g. [19]). Dynamic recalibration appears to be particularly useful for such problems, and a role for the cerebellum is suggested by consideration of the computational complexities of determining target position in eye-centred coordinates of a tactile target delivered to a hand. “If the stimulus is delivered to the finger, the angles of the finger joints, wrist, elbow, shoulder, neck and eyes must be known … a neural implementation of a multi-dimensional lookup table with indexes for all the intervening joint angles could convert stimulus position from body-centred space to eye-centred space” ([19], p.450, p.450). Dynamic coordinate alignment is crucial for motor coordination in multi-jointed animals, and its implementation by the cerebellum could greatly simplify higher-level motor control. One suggestion for future work would be to investigate to what extent the tactile/visual map exemplar could be considered as (or rephrased as) an eye-position/retinotopic.

### Implications for robotics

Finally, dynamic recalibration might also prove useful for biomimetic control schemes in robotics. The adaptive-filter model of the cerebellum has been applied to a number of robot control problems, including plant compensation [48, 49] and the reafference problem [50]. Preliminary results suggest that adaptive-filter based dynamic remapping can be utilised with a robotic platform to improve the accuracy of orienting responses [51]. More generally, the dynamic coordinate transformations referred to above are also required for control of multijoint robots, and it is possible the scheme investigated here could be useful in that context.

## Materials and methods

### Task 1: Calibration of unimodal sensory map

#### Target representation: Writing to the collicular map

Target locations **x**_**d**_ = (*x*_*d*_,*y*_*d*_) were selected randomly from within a two dimensional grid with the limits *x* = −*x*_*t*_ *to x*_*t*_ and *y* = −*y*_*t*_ *to y*_*t*_ where *x*_*t*_ = *y*_*t*_, and *x*_*t*_ was set to 0.75. The target location was transformed into sensor data which was then written into the collicular map, modelled as an *n*_*g*_×*n*_*g*_ square grid spanning *x* = −*x*_*max*_ *to x*_*max*_ and *y* = −*y*_*max*_ *to y*_*max*_, with values *n*_*g*_ = 100 and *x*_*max*_ = *y*_*max*_ = 1.5. Each grid point corresponds to a collicular neuron. The sensor data were generated by a linear sensor model which for a calibrated map gives the sensor data **s**_**d**_ = (*v*_*d*_,*w*_*d*_) which is the accurate sensed position of target **x**_**d**_

$$s_{d}=K\;x_{d}$$ Eq (1)

where **K** is a 2 x 2 matrix that defines the sensor model. It was set in the single-sensor calibration task to [0.8944 0; 0.2739 0.7906].

The sensor data were written into the topographic collicular map to provide a distributed representation of the target location (Fig 1D). Neurons in the map had receptive field centres (*x*_*i*_,*y*_*j*_), so that if only an individual neuron fired, it would produce an orienting response to the real-world location (*x*_*i*_,*y*_*j*_). The response: $g_{x_{i,}y_{j}}(s_{d})$ is the response of the neuron in the map with receptive field centre (*x*_*i*_,*y*_*j*_) to sensor input **s**_**d**_. We write $g_{i,j}(s_{d})=g_{x_{i}{,y}_{i}}(s_{d})$. A 2D elliptical Gaussian function was used to provide the distributed target position. In this representation, the map output estimates the preferred target location **x**_**d**_ from the responses of individual neurons described by a covariance matrix cov [**x**] = *Σ*

$$g_{i,j}(s_{d})=e^{-{0.5((x_{i,j}-x_{d})}^{T}\Sigma^{-1}(x_{i,j}-x_{d}))}$$ Eq (2)

For the unimodal map **Σ** was set to [0.0125 -0.0043; -0.0043 0.0175].

#### Collicular outputs: Reading the collicular map

The collicular output is sent to the motor system, where it generates an orienting response to the estimated position of the target, in this case the actual position of the target **x**_**d**_. Its two components can be calculated as

(xdyd)=∑(rixrjy)gi,j(sd) Eq (3)

where the values $r_{i,j}^{x,y}$ code the position of receptive-field centre of neuron (*i,j*) and vary smoothly across the map (e.g. [52]). For the calibrated map

$$x_{d}=(x_{d},y_{d})=K^{‐1}\;s_{d}$$ Eq (4)

A copy of the distributed neuronal response (Eq 2) is also sent to the cerebellum as mossy-fibre input, where it is processed in the granular layer to produce a coarse coded map carried by the parallel fibres (Fig 1D). An evenly spaced *k* by *k* grid of Gaussian receptive fields, $G_{P}^{n}$ (where *n* denotes the *n*^*th*^ Gaussian in the *k* by *k* grid) was used to coarse code the topographic map. We use *k* = 8, and a grid of slightly overlapping, symmetrical Gaussians for $G_{P}^{n}$ with covariance matrix [0.0352 0; 0 0.0352]. The activity of each grid point was found by multiplying each Gaussian receptive field by the topographic map activity and summing and normalising. The non-normalised activity of each grid point is given as

$$q_{n}=\sum_{i,j}G_{P}^{n}g_{i,j}(s_{d})$$ Eq (5)

The parallel fibre signals are normalised versions of the coarse coded map and given as

$$P_{n}=\frac{q_{n}}{\sum_{k^{2}}q_{n}}.$$ Eq (6)

There are *k*^2^ parallel fibre signals and *P*_*n*_ is the value of the coarse coded, normalised parallel fibre signal at the nth grid point. *P* is a vector containing all parallel fibre signals *P* = [*P*_1_,*P*_2_,….,*P*_*N*_] where *N* is the total number of parallel fibre signals and equal to *k*^2^.

#### Calibration of distorted unimodal collicular map

To produce inaccurate maps, the sensor data were distorted so that the actual value **s**_**g**_ = (*v*_*g*_,*w*_*g*_) written into the collicular map differed from the required value **s**_**d**_ = (*v*_*d*_,*w*_*d*_)

$$s_{g}=A\;s_{d}+a+B\;{s_{d}.}^{2}+C\;{s_{d}.}^{3}$$ Eq (7)

The matrices **A**,**B**,**C**,**a** define the distortion, the symbol ‘.’ indicates an element by element operation. The (inaccurate) target position **x**_**g**_ = (*x*_*g*_,*y*_*g*_) is estimated from the distorted, sensed signal as

$$x_{g}=K^{-1}s_{g}$$ Eq (8)

For the distortion used here the values were: **A** = [1.1 0.1; -0.2 0.9]; **a** = [0.00; -0.2]; **B** = [0–0.05; 0.05 0.1]; **C** = [0.1 0.7; -0.8 0].

The response of individual neurons in the collicular map is given by:

$$g_{i,j}(s_{g})=e^{-{0.5((x_{i,j}-x_{g})}^{T}\Sigma^{-1}(x_{i,j}-x_{g}))}$$ Eq (9)

When the map is distorted, the collicular output sent to the motor system is incorrect, so producing an inaccurate orienting response to location **x**_**g**_ rather than to **x**_**d**_. The components of this response can be calculated as

(xgyg)=∑(rixrjy)gi,j(sg) Eq (10)

where, as previously, the values $r_{i,j}^{x,y}$ code the centre position of neuron (*i*,*j*) and vary smoothly across the map. The corresponding parallel-fibre output is given by:

$$q_{n}=\sum_{i,j}G_{P}^{n}g_{i,j}(s_{g})$$ Eq (11)

### Cerebellar calibration

When the collicular map is not correctly calibrated, the estimated target position **x**_**g**_ will differ from the actual location **x**_**d**_, and the orienting movement will be in error(**e** = **x**_**d**_−**x**_**g**_ = (*e*_*x*_, *e*_*y*_)). The cerebellum receives a corresponding error signal via climbing fibres, a signal assumed here to be signed and two dimensional with axes approximately aligned with horizontal and vertical (x and y directions), which is used to adjust the weights associated with each parallel fibre signal

$$\Delta w_{x}=-\beta \;e_{x}\;P$$ Eq (12)

$$\Delta w_{y}=-\beta \;e_{y}\;P$$

where *e*_*x*_ and *e*_*y*_ are the error components, *P* the coarse coded parallel fibre signals, and *β* is a learning rate here set to 1. The initial value of the weights was zero. The learnt weights were used to bias the map in the *x*—and *y*–directions by generating a cerebellar signal (*δx*, *δy*) corresponding to the sum of the weighted parallel fibre signals

$$\delta x=\sum w_{x}P$$ Eq (13)

$$\delta y=\sum w_{y}P$$

The cerebellar bias signal in effect slides map activity across the map by an amount (*δx*,*δy*).

### Task 2: Combining unimodal sensory maps

#### Target representation: Writing to the collicular maps

Sensor data were written into undistorted unimodal maps using Eq (1). For two independent sensors, two independent sensory maps are generated, with sensor-model parameters **K_1** and **K_2** which were both set to [1 0; 0 1]. The sensor data were written into each map to provide a distributed representation of the target location, as described by Eq (2). The parameters *Σ***_1** and *Σ_***2** were both set to [0.0225 0; 0 0.0225].

#### Target representation: Reading the collicular maps

Map read out however differs when there are multiple maps. The individual unimodal maps do not themselves drive orienting responses, but instead are combined into an overall multimodal map. Neurons with receptive field centres (*x*_*i*_, *y*_*j*_) in the unimodal maps project to the neuron with the same receptive field centre in the multimodal map. In the simplest form of combination, the responses of neurons in this map are given by:

$$G_{i,j}\;(s1_{d},s2_{d})=g_{i,j}(s1_{d})*g_{i,j}(s2_{d})$$ Eq (14)

where * represents an element by element multiplication of the individual unimodal maps. Target position is then estimated from the multimodal map using Eq (3), with the (*x*, *y*) components of the response now calculated with the individual map responses *g*_*i*,*j*_(**s**_**d**_) replaced with the multimodal map responses *G*_*i*,*j*_(**s1**_**d**_,**s2**_**d**_). The output of the multimodal map is sent to the motor system to generate an orienting response to the estimated position of the target, which in the case of undistorted maps will be the actual position of the target **x**_**d.**_

Copies of the distributed neuronal responses in the unimodal maps were also sent to the cerebellum as parallel-fibre inputs (Fig 4). Coarse-coded parallel-fibre signals for each map were generated using Eqs (5) and (6), with the same values for the parameters.

#### Calibration of distorted unimodal maps

When one or both unimodal maps are distorted, the target position **x**_**g**_ estimated by the multimodal map will differ from the actual location **x**_**d**_. With the parameters used here, this estimated target position is the mean of the estimates of the unimodal maps. The inaccurate estimate generates an error in the orienting movement (**e** = **x**_**d**_−**x**_**g**_). We examined three methods for using this error to ensure accurate calibration of the unimodal maps.

In the first method (Method 1), the same error was used to calibrate both unimodal maps, by adjusting the weights of the synapses between parallel fibres and Purkinje cells, so that the cerebellar output to each map biases it to shift the position of peak map activity (Fig 4).

Maps were distorted using the procedure described by Eq (7). Two conditions were run. In the first, the map distortions were arranged so that they cancelled each other out. The parameters for this condition were:

**A_1** = [0.8 0.2; -0.4 1.1] **a_1** = [0; 0] **B_1**= [0 0; 0 0] **C_1**= [0; 0]

**A_2** = [1.15 -0.21; 0.42 0.83] **a_2** = [0; 0] **B_2**= [0 0; 0 0] **C_2**= [0; 0]

Before calibration these distortions produce two inaccurate estimates of target position, **x**_**g**_**_1** and **x**_**g**_**_2** (Eq 8), such that **x**_**g**_**_1** + **x**_**g**_**_2** = 2**x**_**d**_.

The responses of individual neurons in the collicular maps is given by Eq (9). In the second condition, only one of the maps was distorted, with the parameters: **A_1** = [0.75 0.2; -0.4 1.1] **a_1** = [0; 0] **B_1 =** [0.01 0.02; 0.05–0.05] **C_1** = [0; 0].

In both conditions, the output of the two unimodal maps was sent to the bimodal map (Eq 14). The target position **x**_**g**_ estimated by the multimodal map is given by Eq 14. When this estimate differs from the actual location **x**_**d**_, the orienting movement will be in error (**e** = **x**_**d**_−**x**_**g**_), and that error is used as before to alter parallel-fibre synaptic weights (Eq 12) and so generate cerebellar biasing signals (Eq 13). In the case of two maps the learning rule becomes

$$\Delta {w1}_{x}=-\beta 1\;e_{x}\;P$$

$$\Delta {w1}_{y}=-\beta 1\;e_{y}\;P$$ Eq (15)

$$\Delta {w2}_{x}=-\beta 2\;e_{x}\;P$$

$$\Delta {w2}_{y}=-\beta 2e_{y}\;P$$

where *w*1 and *w*2 refer to the vector of parallel-fibre weights for maps 1 and 2 respectively, *e*_*x*_ and *e*_*y*_ are the *x*− and *y*−components of **e**, and *P* the vector of coarse coded parallel fibre signals (combined for multiple sensors), and *β*1 and *β*2 the learning rates. For calibration Method 1, both learning rates were set to 0.25.

The biasing signals for the two maps, ***z***_**1**_ = (*δx*_1_,*δy*_1_) and ***z***_**2**_ = (*δx*_2_,*δy*_2_) were calculated as:

$$\delta x_{1}=\sum {w1}_{x}P$$

$$\delta y_{1}=\sum {w1}_{y}P$$ Eq (16)

$$\delta x_{2}=\sum w2_{x}P$$

$$\delta y_{2}=\sum w2_{y}P$$

These biasing signals adjusted each map’s target estimate as before. The maps were calibrated for 10, 000 trials.

Using the same error signals to train each individual map led to credit assignment problems that did not arise for a single map. For example, sensors can end up miscalibrated in such a way that their errors cancel on average. Furthermore, since the parallel fibre signals to both sets of Purkinje cells contain information from each sensor and so constitute an undifferentiated bus of information, there are problems with parallel fibre cross talk that are also not seen for a single map. Two methods for solving these problems were investigated. The first (Method 2) was gating of error signals.

The same sensor parameters were used as in Method 1. Two conditions were considered: i) the unimodal maps were miscalibrated in such a way that errors cancelled on average so the overall combined map is accurate, and ii) one map was accurate and the other map distorted. Distortion parameters (Eq 7) for both conditions were as in Method 1.

Error gating was introduced so that if a sensor failed to detect a target the error signal was gated (the learning rate for the corresponding sensory map was set to zero for that trial). Failure to detect the target on a given trial was simulated by setting the sensor property **Σ** = [4.5 0; 0 4.5] which effectively gives a very large spread of possible target locations in the sensory map (Fig 4B and 4C). The gating was random such that on average for 1/3 of trials both sensors detect the target so neither error signal was gated, for a further 1/3 of trials sensor 1 was imprecise and the error signal to the corresponding Purkinje cells (Fig 4) gated, and the remaining 1/3 of trials sensor 2 was imprecise and error signal to the corresponding Purkinje cells gated. 10,000 trials were run.

As Fig 4 indicates, the Purkinje cells that bias sensory map 1 receive parallel-fibre signals from map 2, and vice versa. The cross-talk synapses from these parallel fibres potentially allow sensors to calibrate each other, which can result in miscalibration where two miscalibrated maps cancel their effects on estimated target position. To address this problem (Method 3) we introduced independent sensory noise to Eq (1) alongside gating to remove parallel fibre cross talk synapses:

$${s1}_{d}=K\_1\;x_{d}+N(0,\sigma )$$ Eq (17)

$${s2}_{d}=K\_2\;x_{d}+N(0,\sigma )$$

*N*(0,*σ*) indicates Gaussian noise with mean 0 and standard deviation *σ*. Here *σ* was set to 0.005.

The properties of each sensor were again set to **K_1** = **K_2 =** [1 0; 0 1] and **Σ_1** = **Σ_2** = [0.0225 -0.0000; 0.0000 0.0225]. Unimodal map 1 was distorted using Eq (7) with parameters **A_1** = [0.7–0.2; -0.3 0.9]; **a_1** = [0.1; 0.25]; **B_1** = [0 0.0; 0.0 0]; **C_1** = [0; 0];. unimodal map 2 was distorted with parameters **A_2** = [0.8–0.2; -0.1 1.1]; **a_2** = [-0.5; 0]; **B_2** = [0 0.0; 0.0 0]; **C_2** = [0; 0]. The procedure for gating was that used in Method 2, with the nominal learning rate set at 0.25. The system was trained for 15,000 trials.

It should be noted that the method used here for combining single unimodal maps to produce an overall map of target position implements Bayes rule

$$G_{x}(s)=p(x|s_{1},..,s_{n})\propto p(x)p(s_{1},\ldots ,s_{n}|x)\propto p(x)\;\prod p(s_{k}|x)=p(x)\;\prod g_{x}(s_{k})$$ Eq (18)

where **s**_**1**_,..,***s***_***n***_ represents sensory data from each of the n sensors, the prior *p*(**x**) can be interpreted as an attentional search light (for simplicity we assume that the prior for each sensor is unity), and the overall activity calculated by taking the product of the activities in registered maps for each sensor.

### Task 3: Predictive recalibration

Sensory maps can be used for the pursuit of moving targets. We therefore examined whether the proposed role of the cerebellum in calibrating a unimodal sensory map using stationary targets (Fig 1) could be extended to pursuit. For moving targets delays in sensory processing (for example in the retina) become important, because the map no longer has access to the current target location **x**(T+Δ) (where Δ is the delay and T the trial number, Fig 7A) but only to its delayed location **x**(T). In addition the error signal is no longer the difference between current estimated and actual target locations, but between current estimated location and actual location Δ times steps earlier (Fig 7A). To solve this calibration problem the system must learn to predict future target location, hence the term predictive recalibration.

The parallel-fibre signals from map to cerebellum now conveyed temporal information, required for the prediction of target trajectories. The new temporal signals were generated by a bank of fixed temporal filters (Fig 7A). Incorporating fixed filters increases the number of parallel fibre signals and corresponding weights to adjust, but does not change the rest of the algorithm.

#### Target representation: Writing to the collicular map

Whereas in previous tasks target locations **x**_**d**_ = (*x*_*d*_,*y*_*d*_) were selected randomly, here they were specified using a two-dimensional, low-frequency, coloured noise trajectory. The trajectory was made up from a vector of x-values and one of y-values, both with length N, where N = 10,000. To construct these vectors, we used a sampling frequency of 20Hz (so that successive vector values represented target locations 50 msec apart) and first generated values from the uniform distribution on the interval −0.75 to 0.75. Frequencies greater than 0.5Hz were removed to give a low-frequency trajectory. The trajectory was re-scaled to ensure that it reached the limits in x- and y- more to enable correct calibration of the grid at the limits. This rescaling was done using a sigmoid function. An example (14.25secs long) of a target trajectory generated in this way is shown in Fig 7B (shorter example trajectories of 3.75s are given in Fig 7D). The resulting target trajectories are only predictable in the sense that they have some structure derived from filtered white noise.

Sensor data were written into the collicular map as in task 1, with parameters **K =** [0.8944 0; 0.2739 0.7906] and **Σ** = [0.0125 -0.0043; -0.0043 0.0175]. Sensor delay Δ was 5 samples (250 msec).

#### Collicular outputs: Reading the collicular map

The collicular map output was sent to the motor system to generate an orienting response, as for the unimodal map. A coarse coded copy of the neuronal response was also sent to the cerebellum, only now as outlined above it is passed through a set of temporal filters. The normalised signals *P*_*n*_(Eq 6) from the *n*th grid point were passed through fixed filters **G**_**1**_−**G**_**r**_ giving outputs of the form $P1_{n}=G_{1}(P_{n})$.

Here 3 leaky integrator filters were used, with log-spaced time constants T = 0.0500, 0.0707,0.1000 sec. The outputs of these filters were then approximately decorrelated into signals *P*1_*n*_−*P*3_*n*_(where *n* = 1→*k*^2^, the number of parallel fibre signals is increased as a multiple of the number of fixed filters) using a fixed matrix Q as described in Wilson et al. [53]. The overall parallel fibre signals are now given as *P* = [*P*1_1_,*P*1_2_,…*P*1_*η*_,*P*2_1_,*P*2_2_,…*P*2_*η*_,*P*3_1_,*P*3_2_,…*P*3_*η*_] where *η* = *k*^2^.

#### Predictive Calibration of distorted collicular map

The map was distorted as described in Eq 7 with **A** = [1.2 0.2; -0.3 0.9], **a** = [0.0; 0], **B** = [0–0.05; 0.05 0.01], **C** = [0; 0]. The collicular output sent to the motor system was thus incorrect, due both to the distortion (Eq 10) and the delay (Δ = 100 msec). As indicated in Fig 7A, the error was now the difference between the desired target location in the future (at time orient takes place) and the actual target location at each point in time (**e** = **x**_**d**_(Δ+T)−**x**_**a**_(T)).

The cerebellar learning algorithm given in Eqs 12 and 13 remained unchanged, although the number of weights to learn is increased to three times the number in task 1.

For predictive calibration, we used a learning rate of *β* = 5 and a signal trajectory with 10000 data points.

#### One dimensional targets moving with constant velocity

The map calibration algorithm was also applied to targets moving with a constant velocity in one dimensional space (e.g. the target location **x**_**d**_ = (*x*) now has a single dimension). In this simulation, no distortion was applied, but signals were delayed by 100 samples. The sampling frequency was *dt* = 5ms.

The map was coarse coded using a grid of 8 Gaussians, with evenly spaced centres. This was then filtered using a bank of three leaky integrator filters, with log-spaced time constants T = 0.0500, 0.0707, 0.1000 to give a total of 24 parallel fibre signals. The sensor parameters were **K =** 0.1094 and **Σ** = 0.7559. For calibration, we used a learning rate of *β* = 25. Distinct constant velocity targets with positive and negative velocities of 0.125, 0.5, 1 and 2ms^-1^ were used as trajectories.
